# Isolation, Characterization, and Anti-Idiopathic Pulmonary Fibrosis Activity of a Fucoidan from *Costaria costata*

**DOI:** 10.3390/molecules28114343

**Published:** 2023-05-25

**Authors:** Sijie Wei, Lihua Geng, Haoyu Yu, Jing Wang, Yang Yue, Quanbin Zhang, Ning Wu

**Affiliations:** 1 CAS and Shandong Province Key Laboratory of Experimental Marine Biology, Center for Ocean Mega-Science, Institute of Oceanology, Chinese Academy of Sciences, Qingdao 266071, China; weisijie20@mails.ucas.ac.cn (S.W.);; 2 University of Chinese Academy of Sciences, Beijing 101408, China; 3 Drugs and Bioproducts, Qingdao National Laboratory for Marine Science and Technology, Qingdao 266237, China; 4 Nantong Zhongke Marine Science and Technology Research and Development Center, Nantong 226682, China

**Keywords:** *Costaria costata*, fucoidan, idiopathic pulmonary fibrosis

## Abstract

Pulmonary fibrosis is a chronic, progressive, and fatal disease of the interstitial lung. There is currently a lack of efficient therapy to reverse the prognosis of patients. In this study, a fucoidan from *Costaria costata* was isolated, and its anti-idiopathic fibrosis activity was investigated both in vitro and in vivo. The chemical composition analysis showed that *C. costata* polysaccharide (CCP) consists of galactose and fucose as the main monosaccharides with a sulfate group content of 18.54%. Further study found that CCP could resist TGF-β1-induced epithelial-mesenchymal transition (EMT) in A549 cells by inhibiting the TGF-β/Smad and PI3K/AKT/mTOR signaling pathways. Moreover, in vivo study found that CCP treatment alleviated bleomycin (BLM)-stimulated fibrosis and inflammation in mice lung tissue. In conclusion, the present study suggests that CCP could protect the lung from fibrosis by relieving the EMT process and inflammation in lung cells.

## 1. Introduction

Idiopathic pulmonary fibrosis (IPF) is characterized by a diffuse interstitial fibrotic change in the lung parenchymal [1]. Because of its rapid deterioration and high lethality, it is considered a difficult pulmonary disease by the World Health Organization [2]. The pathological symptom of IPF in the early stages is alveolar inflammation involving fibroblast proliferation and massive extracellular matrix (ECM) deposition. Eventually, this abnormal accumulation of collagen leads to pulmonary fibrosis. Several signal pathways have been implicated in the pathogenesis of IPF. Among these, the TGF-β/Smad signal pathway plays an essential role.

The streamer kelp *C. costata* is in the order *Laminariales*, family *Costariaceae* [3], which is an annual plant found mainly in Troits Bay in the Sea of Japan and in Russia [4]. *C. costata* has an undivided blade over 30 cm in width and about 150 cm long and grows to its largest size in June [5]. It has been used as edible seaweed for a long time in Japan and as a nutritious foodstuff for *Abalone*, *Haliotis*. In recent years, *C. costata* has been successfully introduced to the coast of Dalian in northern China for captive breeding. 

Fucoidan is a water-soluble polysaccharide with a sizable quantity of fucose and sulfate groups. It is mainly derived from brown algae and is also found in marine invertebrates [6]. Kylin isolated fucoidan for the first time in 1913 and named it “fucoidin” [7]. The structure and function of fucoidan vary according to the species of brown algae and extraction techniques. For instance, January evaluated three different fucoidan extraction techniques: hot water extraction, salt extraction, and acid extraction. It was found that hot water extraction provides the maximum yield of fucoidan [8].

The fucoidan derived from *C. costata* has a wide range of biological activities, such as anti-hyperlipidemia [9], anti-skin photoaging [3], and anti-CCl_4_-induced liver injury [4]. However, there is no relative research concerning the anti-idiopathic pulmonary fibrosis activity of fucoidan from *C. costata*, so in the present study, we isolated fucoidan (CCP) from *C. costata* using the hot water extraction method, analyzed the chemical composition of CCP, and firstly evaluated its anti-idiopathic fibrosis activity in both an in vitro cell model and in vivo mice model. 

## 2. Results and Discussion

### 2.1. Chemical Properties of CCP

**Figure 1:**
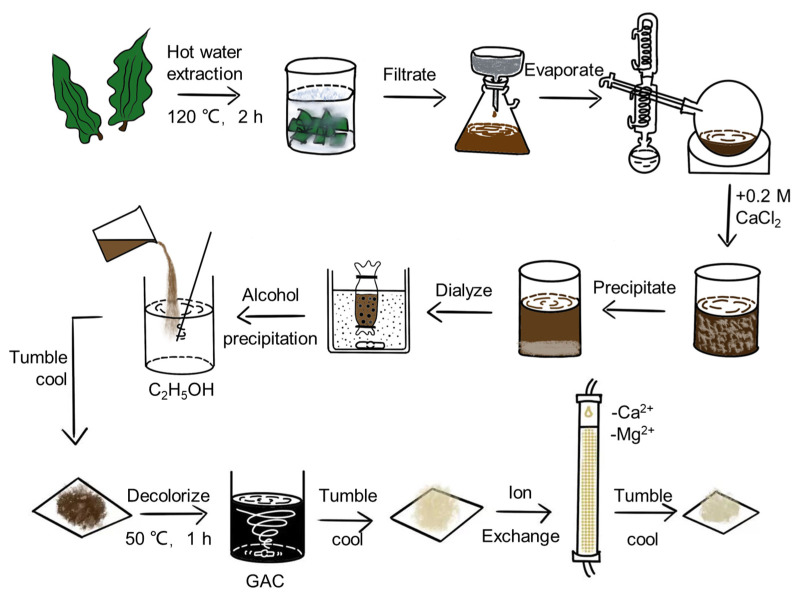
The extraction and purification process of CCP.

Ethanol precipitation is currently the most commonly used method to extract polysaccharides because polysaccharides are soluble in water but insoluble in organic solvents such as alcohol. Specifically, ethanol is added to the extraction solution to reduce the dielectric constant so that the polysaccharides can be precipitated by dehydration [10]. The main process of alcohol precipitation is divided into crushing, leaching, and alcohol precipitation, among which crushing and leaching have a great influence on the yield, quality, and economy of obtaining polysaccharides [11]. In addition to mechanical crushing, novel techniques have emerged for the extraction of fucoidan, including ultrasound, microwave, and enzyme-assisted extraction [12]. Hot water extraction, dilute acid extraction, and dilute alkali extraction are the main leaching methods [13,14]. Therefore, for convenience and economy, we chose a combination of mechanical crushing, hot water extraction, and ethanol precipitation. The CCP extraction and purification process is shown in Figure 1. The yield of CCP was 6.90% by hot water extraction, CCP was decolorized using activated carbon, and the absorbance at 580 nm was measured before and after decolorization; the decolorization rate was calculated to be 82.86% using the following equation (Equation (1)):

(1) $$\mathrm{Decolorization}\;\mathrm{rate}=\frac{A_{Before\;decolorization}-A_{After\;decolorization}}{A_{Before\;decolorization}}\times 100\%$$

Typically, before bioactivity tests, the polysaccharide mixture is subjected to one or more purification processes to further enrich the extracts with the polysaccharides of interest [15]. Therefore, CCP was treated with cation exchange column chromatography to remove Ca^2+^ and Mg^2+^. The Ca^2+^ and Mg^2+^ contents before and after ion exchange were detected using an elemental analyzer, yielding calcium and magnesium ion removal rates of 99.2% and 95.5%, respectively, calculated using the following equation (Equation (2)):

(2) $$\mathrm{Ion}\;\mathrm{removal}\;\mathrm{rate}=\frac{C_{Before}-C_{After}}{C_{Before}}\times 100\%$$

**Figure 2:**
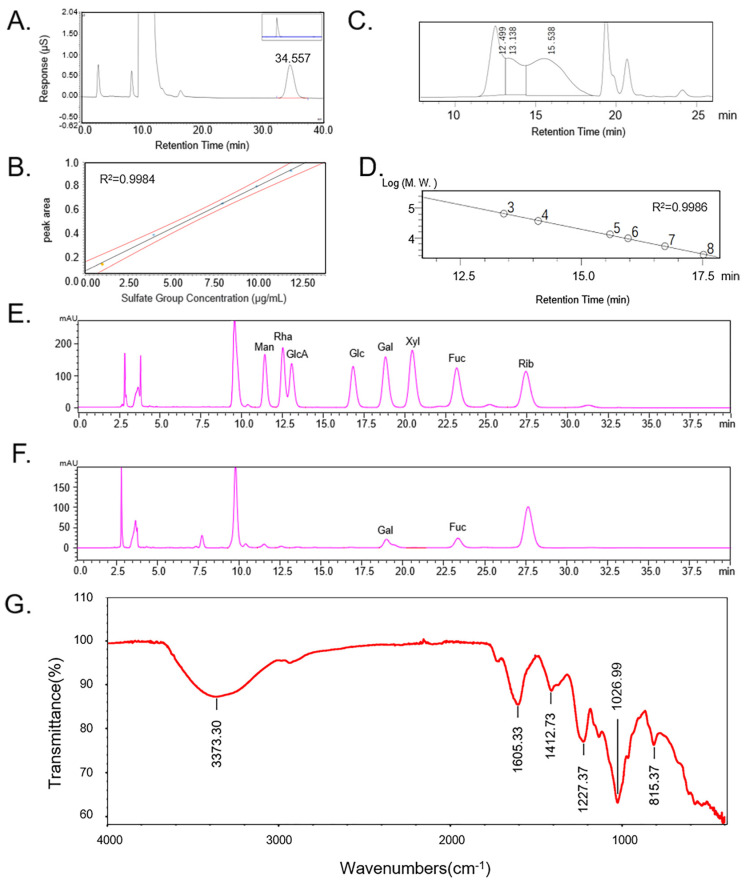
Chemical properties of CCP. (**A**) Determination of the sulfate group content of CCP by ion chromatography. (**B**) Standard curves for the determination of the sulfate group content (1 μg/mL, 2 μg/mL, 4 μg/mL, 6 μg/mL, 8 μg/mL, 10 μg/mL, 12 μg/mL). (**C**) Chromatogram for determination of the molecular weight of CCP. (**D**) Standard curve for molecular weight determination. 3: 64650 Da, 4: 36800 Da, 5: 13150 Da, 6: 9750 Da, 7: 5250 Da, 8: 2700 Da. (**E**) Chromatogram of standards for monosaccharide composition analysis (mannose (Man), rhamnose (Rha), glucuronide (GlcA), glucose (Glc), galactose (Gal), xylose (Xyl), fucose (Fuc). (**F**) Chromatogram of CCP for monosaccharide composition analysis. (**G**) ATR−FTIR spectrum of CCP.

Proteins and phenolic compounds can also show some beneficial biological activity in vitro and in vivo, and they are usually mixed with polysaccharides together during polysaccharide extraction [16]. Prior to testing for biological activity, tests were carried out on the protein content and total phenolic content to exclude any effect they might have on the biological activity. The protein content of CCP was 1.38% by BCA; phenols were not detected in CCP. In addition, the sulfate group content was 18.54% by ion chromatography (Figure 2A,B) analysis. The total sugar content of CCP was 99.86%, and the fucose content was 14.84% using fucose as standard, the glucuronide content of CCP was 13.20% using glucuronide as standard, and the molecular weight of CCP was 149,846 Da by HPLC (Table 1). The yield was calculated by Equation (3). Sulfate content, total sugar content, fucose content, GlcA content, and protein content were calculated by standard curves, respectively, which represent their content in CCP. 

The molecular weight of CCP was calculated as 149.8 kDa according to the standard curve (Figure 2C,D). CCP mainly contains Fuc and Gal with a molar ratio of 1:0.906, which indicates that CCP is a sulfated polymer of galactose and fucose (Figure 2E,F). 

The infrared characteristics of CCP are shown in Figure 2G. There is a large, wide peak between 3100 and 3500 cm^−1^, assigned as the vibration absorption of -OH. The variable angle vibration absorption of the amide group N-H is at 1605 cm^−1^. The variable angle vibration absorption of C-H is at 1412 cm^−1^. The sharp and strong absorption peaks at about 1227 cm^−1^ is the S=O vibration absorption; there is characteristic absorption of sulfated or amino groups. In the fingerprint region, the sharp and weak peak at 815 cm^−1^ is classified as C-O-S stretching vibration absorption peak, suggesting that the sulfate substitution may be at the C-2 position [17]. The results show CCP is an acidic polysaccharide.

### 2.2. CCP Attenuated TGF-β1-Induced EMT in A549 Cells

**Figure 3:**
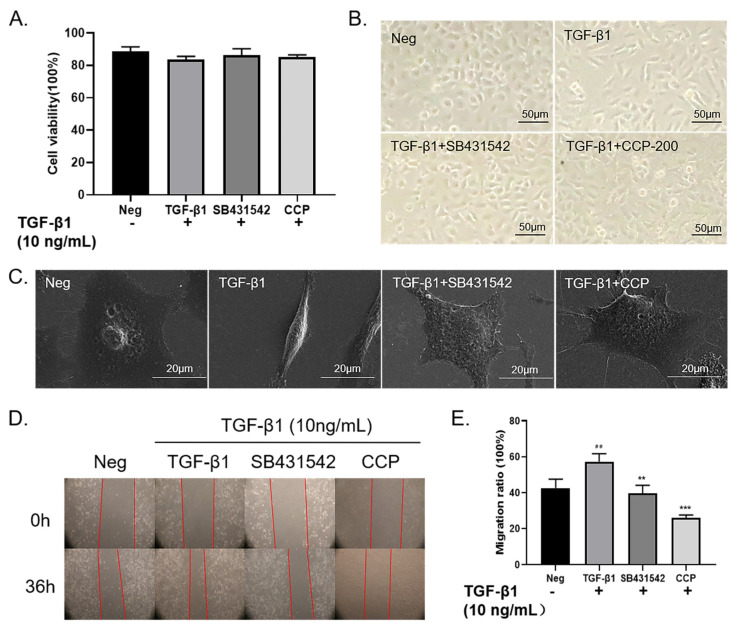
CCP attenuated TGF−β1−induced EMT-like phenotypic change in A549 cells. Cell cytotoxic assay (**A**). TGF−β1 (10 ng/mL), SB431542 (4 μM), CCP (200 μg/mL) Observation of cell morphology under an ordinary light microscope (**B**) and scanning electron microscope (**C**), Migration of A549 cells in the Neg, TGF−β1, SB431542, and CCP groups when A549 cells were treated for 0 h and 36 h, respectively. Red lines represent cell scratch boundaries. Images were taken with a 4× objective (**D**). Analysis of migration rate (%) compared to TGF−β1 group (**E**) ** *p* < 0.01, *** *p* < 0.001, compared to control (Neg): ## *p* < 0.01. All values are mean ± standard error and were pooled from five independent experiments.

EMT is the terminal polarized epithelial to mesenchymal transition: injured epithelial cells transformed into fibroblasts and myofibroblasts, with the cells gaining migratory properties and losing the regeneration capacity of normal epithelial cells after transformation. TGF-β1 is known to play a critical role in the EMT process. Several studies have used TGF-β1 as an inducer for transforming polygonal epithelial cells into spindle-shaped fibrocytes [18]. In our study, A549 cells were induced using 10 ng/mL TGF-β1 and administered with CCP (200 μg/mL) or SB431542 (4 μM), followed by 36 h of incubation. After this treatment, A549 cells were subjected to cytotoxicity assay using MTT. The results of our previous study showed that there was no significant change in cell survival in TGF-β1, TGF-β1 + SB431542, and TGF-β1 + CCP treated groups (Figure 3A). Compared with the blank control group, the cell viability of the TGF-β1 induction group, TGF-β1+ SB431542 group, and TGF-β1 + CCP induction group decreased by 5.24%, 2.37%, and 3.64%, respectively.

As Figure 3B shows, the morphology of the TGF-β1-induced cells changed to a narrow spindle shape with reduced intercellular contacts compared to the normal morphology of the negative control A549 cells, which were epithelial-like and polygonal. In terms of cell morphology, TGF-β1 induced not only changed cell morphology from the normal epithelial-like polygonal shape to the typical narrow shuttle shape but also reduced intercellular contacts. However, compared to the TGF-β1-induced group, 4 μM SB431542 or 200 μg/mL CCP treatment of cells restored intercellular contacts to normal polygonal shape and reproduced intercellular contacts [19]. We performed transmission electron microscopy (Figure 3C) to further validate the ameliorative effect of CCP on TGF-β1-induced EMT phenotypic alterations. The results revealed after the treatment of TGF-β1, the tumor tissue of A549 changed from the original prism to a long fusiform. However, the cell morphology resumed diamond shape in SB431542 and CCP-treated cells. These images illustrated that CCP attenuates TGF-β1-induced EMT-like phenotypic changes in A549 cells. 

Growing evidence suggests that EMT causes significant morphological changes in differentiated epithelial cells and enhances their motility and invasive ability [20,21]. TGF-β1 induces cell migration and cell dedifferentiation, and it also regulates the expression of ECM components, such as fibronectin, elastin, and collagen [22]. To investigate whether CCP can inhibit EMT, the migration and invasion ability of cells was assessed by wound-healing assay (Figure 3D,E). The results indicated that cell migration was dramatically increased in TGF-β1 induced cells compared with A549 cells, and SB431542 or CCP treatments were able to inhibit the enhanced migration ability of EMT to varying degrees. We demonstrated that CCP could inhibit TGF-induced EMT in A549 cells from the perspective of cell migration.

### 2.3. CCP Attenuates EMT by Inhibiting Both TGF-β/Smad and PI3K/AKT/mTOR Signaling Pathway

**Figure 4:**
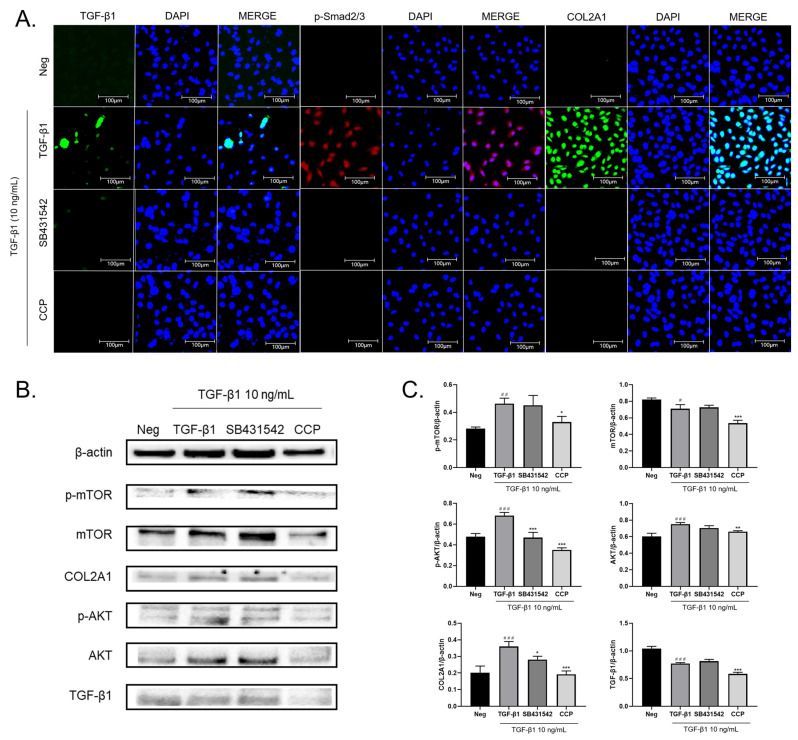
CCP impaired the TGF-β1-induced EMT process in A549 cells by inhibiting both TGF-β/Smad and PI3K/AKT/mTOR signaling pathways. (**A**) The protein levels of TGF-β1, p-Smad2/3, and COL2A1 from A549 cells treated with TGF-β1 (10 ng/mL), TGF-β1 plus SB431542 (4 μM), TGF-β1 plus CCP (200 μg/mL) or negative control (Neg) were determined by immunofluorescence, with scale lengths representing 50 μm. All values are mean ± standard error and were pooled from three independent experiments. (**B**) The protein levels of p-mTOR, mTOR, COL2A1, p-AKT, AKT, and TGF-β1 from A549 cells treated with TGF-β1 (10 ng/mL), TGF-β1 plus SB431542 (4 μM), TGF-β1 plus CCP (200 μg/mL) or negative control (Neg) were determined by western blot, and analyzed by Image J (**C**), compared to TGF-β1 group: * *p* < 0.05, ** *p* < 0.01, *** *p* < 0.001, compared to control (Neg): # *p* < 0.05, ## *p* < 0.01, ### *p* < 0.001.

Generally speaking, the TGF-β1-induced EMT pathway can be divided into two types, Smad-dependent and Smad-independent paths, of which the TGF-β1/Smad signal path is the most studied [23,24]. TGF-β1 can interact with TGF-β receptors I and II (TGFβRI and TGFβRII) to promote the phosphorylation of Smad2/3 [25]. Phosphate-modified Smad2 and/or Smad3 bind to Smad4, then transport the conjugate into the cell and regulate EMT-related transcriptional molecules, such as Snail1, Twist1, and Slug [26,27]. In addition, non-Smad pathways have also been involved in the development of EMT [28]. For example, activation of the AKT pathway has important implications for TGF-β1-dependent EMT: in Wu’s study [29], TGF-β1 can activate PI3K/AKT/mTOR and upregulate the expression of COL2A1. Our findings are consistent with this. Using immunofluorescence, we found that TGF-β1 induction significantly increased the expression levels of TGF-β1, p-Smad 2/3, and COL2A1 in A549 cells, while SB431542 or CCP treatment decreased the expression of these cytokines (Figure 4A). It is suggested that CCP may promote the occurrence of EMT in lung cancer through TGF-β/Smad and its downstream molecular mechanism. 

As shown in Figure 4B,C, TGF-β1 induction significantly upregulated the expression of TGF-β1, COL2A1, mTOR, p-mTOR, AKT, and p-AKT. CCP administration significantly inhibited the elevated protein expression caused by TGF-β1, while the reduction effect of SB431542 administration was not statistically significant to p-mTOR, mTOR, and AKT. This result is consistent with immunofluorescence results, indicating that CCP alleviates the TGF-β1-induced EMT phenomenon in A549 cells by inhibiting the PI3K/AKT/mTOR and the TGF-β1/Smad pathways.

### 2.4. CCP Attenuated Lung EMT Phenotype in a BLM-Induced Mice Model

**Figure 5:**
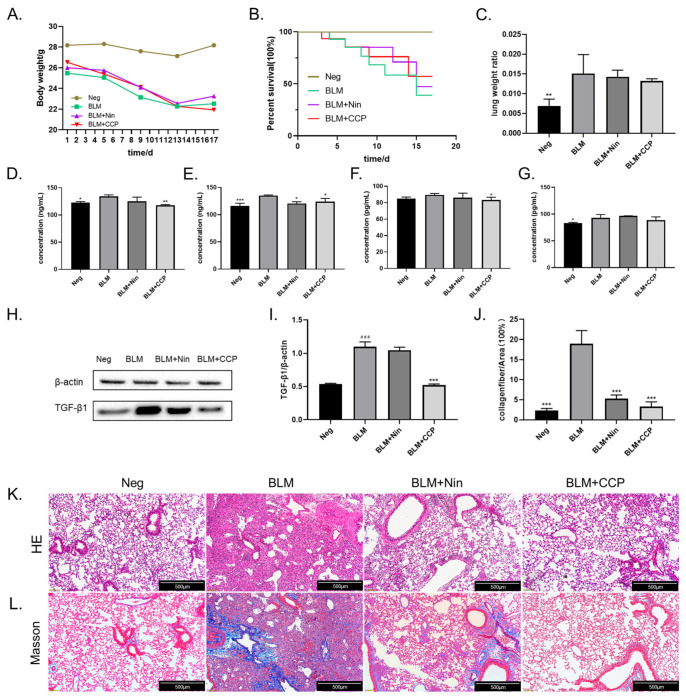
CCP has a palliative effect on bleomycin (BLM)-induced pulmonary fibrosis in mice. (Neg) negative control group, (BLM) BLM (5 mg/kg)-induced group, (BLM + Nin) BLM (5 mg/kg) + Nin (50 mg/Kg)-induced group, and (BLM + CCP) BLM (5 mg/kg) + CCP (200 mg/kg)-induced group. Body weight (**A**), survival rate (**B**), lung-to-weight ratio (**C**), compared to BLM group: ** *p* < 0.01, concentration of TGF-β1 in lung flushing fluid (**D**), concentration of TGF-β1 in serum (**E**), concentration of IL-6 in lung flushing fluid (**F**), concentration of IL-6 in serum (**G**) were determined. The protein levels of TGF-β1 from mice treated with BLM, BLM + Nin, BLM + CCP, or negative control (Neg) were determined by western blot (**H**) and analyzed by Image J (**I**), compared to BLM group: *** *p* < 0.001. compared to control (Neg): ### *p* < 0.001. Collagen fiber to area ratio in Masson’s trichrome staining (**J**) was quantitatively calculated using Image J. Representative images of HE-stained (**K**) and Masson’s trichrome-stained (**L**) lung sections from mice were shown. Scale lengths in the diagram represent 500 μm. Data are shown as the means ± SEM. * *p* < 0.05, ** *p* < 0.01, *** *p* < 0.001.

Intratracheal instillation of bleomycin (BLM) for rapid induction of pulmonary fibrosis (PF) is the classic method for establishing a well-defined PF mouse model [30,31], and we established a fibrosis model in this way. The mice decreased in weight between 1–13 days post-operatively, with the BLM-induced mice losing the most weight and the CCP-induced mice declining the least. The weight of all groups except the CCP group ceased to drop after day 13. In the control group, the weight remained at normal levels. This suggests that the surgery caused a degree of weight loss in the mice, which was mitigated by CCP treatment. In terms of survival, the negative group (Neg) without any treatment had a 100% survival rate, the BLM-induced mice (BLM) had the lowest survival rate, the Nin-administration group (BLM + Nin) had the next lowest survival rate, and the CCP-treated mice had the highest survival rate among the different groups of surgically treated mice (Figure 5B). In addition, we also calculated the lung-to-weight ratio of mice after dissection. We found that due to the inflammatory response and fluid infiltration in the lungs, BLM induction caused a significant increase in the lung-to-weight ratio of mice compared to the Neg group, while the Nin and CCP treatment groups decreased the weight of the lungs (Figure 5C).

Overexpression of TGF-β1 in the lung tissue of IPF patients is often an important feature. We examined the level of TGF-β1 in the alveolar lavage fluid of mice by ELISA (Figure 5D). We found that the expression of TGF-β1 was increased in BLM-induced mice and reduced in Nin and CCP-treated mice compared with the BLM group, and the expression of TGF-β1 in serum showed similar results (Figure 5E). The development of IPF is often accompanied by an inflammatory response, and it has been shown that IL6-JAK2-STAT3/STAT1 is a key mechanism for the effective treatment of IPF. This treatment is associated with an induced inflammatory response against IL-6 [32]. We examined the levels of IL-6 in the alveolar perfusate and serum of mice, respectively, and showed that compared with BLM-induced mice, CCP significantly reduced their IL-6 expression (Figure 5F,G). The expression of TGF-β1 in mouse lung tissue was determined by Western blot (Figure 5H,I). The results showed that the expression of TGF-β1 was significantly increased in the BLM group; Compared with the BLM, the mice in the Nin administration groups showed lower expression levels of TGF- β1, but not statistically significant; CCP treatment significantly reduced the expression levels of TGF- β1 in lung tissues. This result suggests that CCP administration alleviates lung fibrosis in mice by reducing the expression level of TGF-β1.

To evaluate the histopathological changes of lung tissue, we performed HE staining and Masson staining on mouse lung sections 17 days after BLM injection. The HE staining image is shown in Figure 5K. The negative group showed intact alveolar space structure as well as normal alveolar compartments thickness. Our study found that BLM can cause severe alveolar and interstitial damage accompanied by inflammation and severe pulmonary fibrotic lesions. After CCP and Nin treatment, the pathological changes in the lung were improved. There were less cell hyperplasia and less damage to alveolar areas and lung structures. The results of Masson staining (Figure 5L) indicate that BLM injection resulted in excessive deposition of mature collagen in the lungs of mice. The collagen deposition was significantly reduced after CCP and Nin treatment compared with the BLM group, but there was still a small amount of collagen found in the Nin group. Quantitative analysis of the Masson staining results (Figure 5J) yielded that, compared to the BLM group, the Nin and CCP administration groups reduced the percentage of collagen by 13.67% and 16.01%, respectively.0

## 3. Materials and Methods

### 3.1. Reagents and Chemicals

Sulphuric acid (95~98%), sodium hydroxide (≥99.0%), hydrogen peroxide (30%), phenol (≥99.0%), ethanol (95%), and hydrochloric acid (12 M) were purchased from Sinopharm (Shanghai, China); RPMI medium was purchased from Hyclone (Logan, UT, USA); 1-phenyl-3-methyl-5-pyrazolone (PMP), 3-(4, 5-dimethylthiazol-2-yl)-2, 5-diphenyltetrazolium bromide (MTT) reagent, standards for monosaccharide composition analysis, dimethylsulfoxide (DMSO), and trifluoroacetic acid (TFA) were purchased from Sigma-Aldrich (St. Louis, MO, USA); Fetal bovine serum (FBS) was purchased from Biological Industries (Israel); Enzyme-linked immunosorbent assay (ELISA) kit of interleukin-6 (IL-6) production analysis was obtained from Mlbio (Shanghai, China); The total phenolic assay (TPA) kit was provided by Boxbio (Beijing, China) and the bicinchoninic acid assay (BCA) kit was provided by Solarbio (Beijing, China); Transforming growth factor beta 1 (TGF-β1) was provided by GenScript (Nanjing, China); TGF-β1 inhibitor SB431542 was obtained from Beyotime (Nantong, Jiangsu, China); Bleomycin (BLM) was obtained from Nippon Kayaku (Takasaki, Japan); Nintedanib (Nin) was purchased from Yuanye Bio-Technology (Shanghai, China); Griess reagent, Hypersensitive reaction and pathogenicity gene (HRP) conjugated goat anti-mouse immunoglobulin G (IgG) secondary antibodies and 4′,6-diamidino-2-phenylindole (DAPI) were provided by Beyotime (Nantong, Jiangsu, China); HRP conjugated goat anti-rabbit IgG secondary antibodies and antibodies to phosphorylated Smad2/3 (p-Smad2/3), phosphorylated AKT (p-AKT), and phosphorylated m-TOR (p-mTOR) were purchased from Affinity (Chicago, IL, USA); Antibodies to collagen II alpha 1 (COL2A1), AKT, mTOR, and TGF-β1 were purchased from Santa Cruz (Dallas, TX, USA).

### 3.2. Costaria Costata Polysaccharides (CCP) Extraction and Analysis

#### 3.2.1. CCP Extraction

*C. costata* was purchased from Hokkaido, Japan, in 2017. To isolate the crude CCP from *C. costata*, a hot water extraction method was utilized [13,14]. Briefly, the *C. costata* fragments were soaked in hot water at a ratio of 1:10 and heated in the autoclave for 120 min without stirring at 120 °C at high pressure. After filtering residue out and condensing to 2 L by spin evaporation, CaCl_2_ was added to the concentrate with a final concentration of 0.2 M to produce the precipitated sodium alginate (SA), and the filtrate was dialyzed for 24 h using a 12,000 Da dialysis bag. Then we poured the filtrate into three times the volume of ethanol for alcohol precipitation and dried the resulting precipitate with high-temperature baking lamps to get crud CCP. Crude CCP was resolved into a 2% solution, then added 4% granular activated carbon (GAC), and this mixture was stirred at 50 °C for an hour to reduce the pigment. After this, the calcium and magnesium ions were removed using a cation exchange column to purify CCP. The yield rate of CCP was calculated by following formula (Equation (3):

(3) $$\mathrm{Yield}\;\mathrm{rate}=\frac{\mathrm{Dry}\;\mathrm{weight}\;\mathrm{of}\;\mathrm{CCP}}{\;\mathrm{Dry}\;\mathrm{weight}\;\mathrm{of}\;Costaria\;costata}\times 100\%$$

#### 3.2.2. Chemical Analysis

The glucuronide content of CCP was determined using the carbazole colorimetric method [33]. The total sugar content of CCP was assayed by the phenol-sulfuric acid colorimetric method [34], with fucose as the standard. The cysteine hydrochloride method was used to determine the fucose content [35]. The protein content of CCP was determined using the Bicinchoninic Acid (BCA) Assay kit (Beijing Solarbio Science & Technology Co., Ltd., Beijing, China). Using ion chromatography, CCP was eluted with 4.5 mmol/L Na_2_CO_3_ and 0.8 mmol/L NaHCO_3_ mixture solution at a flow rate of 1.0 mL·min^−1^ by IC SI-52 4E column (Dionex IonPac AS 23-4 μm, diameter 4 mm) at 45 °C to determinate the sulfate group content. 

#### 3.2.3. Determination of CCP-Molecular Weight

The mass-averaged molar mass of CCP was determined using the high-performance gel permeation chromatography-multiple angle laser light scattering (HPGPC-MALLS) method. CCP was eluted with 0.1 mol/L Na_2_SO_4_ using a TSK-Gel 3000PWXL (7.8 mm × 300 mm) column from TOSOH, Tokyo, Japan, and then monitored at 40 °C using a refractive index detector. To plot the standard curve, eight dextroses (64,650, 36,800, 13,150, 9750, 5250, and 2700 Da) were used as standards. 

#### 3.2.4. Monosaccharide Determination

The monosaccharide composition of CCP was measured using pre-column derivation reversed-phase high-performance liquid chromatography (RP-HPLC) [36]. CCP (5 mg/mL) was hydrolyzed by 2 mol/L trifluoroacetic acid (TFA) for 4 h at 110 °C and then neutralized using 2 mol/L NaOH. Samples or standards were added with ribose as internal standard, respectively, and reacted with 0.5 mol/L PMP for 30 min at 70 °C under alkaline conditions, then neutralized using hydrochloric acid after derivatization. The derivatives were extracted by chloroform, and high-performance liquid chromatography (HPLC) analysis was performed with the chromatographic condition at 40 °C, using a C18 reverse separation column (InertSustain, Japan, 4.6 mm × 150 mm) and an SPD-20A UV-detector (Shimadzu, Kyoto, Japan) (245 nm), the flow rate was 1 mL/min, and the lysate consisted of 83% 0.1 M KH_2_PO_4_ buffer and 17% acetonitrile. To plot the standard curve, l-fucose (Fuc), d-galactose (Gal), d-mannose (Man), d-glucuronic acid (GlcA), l-rhamnose (Rha), d-glucose (Glc), d-ribose (Rib), and d-xylose (Xyl) were used as standards. We calculated each monosaccharide content using the ratio of peak area to the molecular weight of monosaccharides. 

#### 3.2.5. Attenuated Total Refraction-Fourier Transform Infrared (ATR-FTIR) Spectroscopy

The functional groups of CCP were identified by ATR-FTIR using a Nicolet iS 10 FT-IR spectrometer (Thermo Fisher, Waltham, MA, USA) in the wave number range of 400 to 4000 cm^−1^.

### 3.3. Cell Culture and Treatment

Human non-small cell lung cancer cells (A549 cells) were purchased from the Shanghai Institute of Biological Sciences Resource Center, Chinese Academy of Sciences. A549 cells were cultured in RPMI 1640 medium supplemented with 10% FBS and 5% CO_2_. 

After cell apposition, TGF-β1 was added to all cell groups except the negative control group at a final concentration of 10 ng/mL, the SB431542 was added to the positive control group to make its final concentration 4 μM, and CCP (200 mg/mL) was added to the experimental group. After administration, the cells were incubated at 37 °C and 5% CO_2_ for 36 h. 

### 3.4. Animal Models and Drug Treatment

Male C57BL/6J mice at 6–7 weeks of age were obtained from Viewsolid Biotech (Beijing, China). Animals were housed at 23 ± 2 °C with a 12 h dark/light cycle. All animals had free access to food and water and were humanely cared for throughout the study. All animals were cared for in accordance with the National Institute of Health Guide for the Care and Use of Laboratory Animals, and the Experimental Design of this study was approved by the Animal Experimentation Ethics Committee of the Institute of Oceanography, Chinese Academy of Sciences (CTEC-2022-02-01). 

Forty-eight male mice were divided equally into four groups, namely (1) negative control group (Neg), (2) BLM group, (3) BLM + Nin (50 mg/kg), and (4) BLM + CCP (200 mg/kg) group. In detail, 5 mg/kg bleomycin sulfate (S1214, Selleck, Japan) was injected into the trachea of the mice, and the mice in the negative control group were injected with an equal amount of normal saline. One day after the BLM injection, the BLM + CCP and BLM + Nin groups were given CCP 200 mg/kg or Nin 50 mg/kg once a day orally, respectively. The BLM and negative control groups were given the same amount of saline. Mice in each group were sacrificed after being over-anesthetized on the 17th day after surgery.

### 3.5. Cellular Morphology Analysis

After treatment, cell morphological changes in A549 cells were observed using an inverted microscope (OLYMPUS-CKX41, Olympus Corporation, Tokyo, Japan). Before scanning electron microscopy analysis, cell samples were prepared by dehydration in an alcohol gradient, and then cells were observed under scanning electron microscopy (Hitachi-S-3400N, Hitachi Company, Tokyo, Japan). 

### 3.6. Cell Cytotoxicity Assay

Assessment of A549 cell viability using MTT. In detail, A549 cells were treated with trypsin and then inoculated into 96-well plates at a concentration of 1 × 10^4^ cells/well and incubated at 37 °C, 5% CO_2_. Cells were treated as Section 3.3 described; after 36 h of incubation, 10 µL MTT dye was then dropped into each well, and the plate was carefully vortexed and incubated at 37 °C for 4 h at a concentration of 5% CO_2_. The solution was then gently aspirated from the wells, 150 μL of DMSO was added to each well, and the absorbance was read at 490 nm after 10 min of shaking at room temperature [37]. 

The following formula was used to calculate the percentage of cell viability (Equation (4)):

(4) $$\mathrm{cell}\;\mathrm{viability}=\frac{\mathrm{Mean}\;\mathrm{OD}\;\mathrm{of}\;\mathrm{individual}\;\mathrm{Test}\;\mathrm{Group}}{\mathrm{Mean}\;\mathrm{OD}\;\mathrm{of}\;\mathrm{control}\;\mathrm{Group}\;}\times 100\%$$

### 3.7. Cell Wound-Healing Assay

Cells were incubated in six-well plates for the cell wound healing assay. After opposition, the cells were cut off with the top of the straw, and the fragments were removed with a cleaning solution. Then cultured with a new medium of 1% FBS and administered as described in Section 3.3 incubated for 36 h. Cell migration images were taken at 0 and 36 h. By randomly measuring the incision length at the same location at two time points, 0 h and 36 h. Incision length was used to quantify the healing index of the incision by Image J, determined as a percentage. 

### 3.8. HE and Masson Staining

Lung tissue sections were stained with hematoxylin-eosin (HE) and Masson (Servicebio, Wuhan, China) as a means of assessing the degree of pulmonary fibrosis in each group. To assess the extent of pulmonary fibrosis, stained paraffin sections were observed under a light microscope, and 5 arbitrarily selected areas were photographed and recorded. Quantitative score analysis was performed using Image J and averaged.

### 3.9. Immunofluorescence Staining

A549 was planted on glass and cultured in vitro for 36 h, as mentioned above. It was cured with 4% formaldehyde for 15 min, washed with PBS 3 times, and impregnated with 0.01% TritonX-100 for 5 min. The cell was then sealed with 1% bovine serum albumin for 60 min at room temperature; then, the prepared cells were incubated with TGF-β1, COL2A1, or p-Smad 2/3 diluted at 1:1000 at 4 °C overnight. Subsequently, In the mild and dark part of the room, allospecific secondary antibodies (AlexaFluor594-AffiniPure sheep anti-rabbit or FITC labeled sheep anti-mouse) were stained 1 h later. The staining time of that DAPI method was 5 min, then cells were rinsed with PBS three times after that [38]. Finally, an anti-fluorescence quencher (Sigma-Aldrich, St. Louis, MO, USA) was added. A laser scanning confocal microscope (Zeiss, Jena, Germany) collected the images. 

### 3.10. Western Blot Analysis

Fresh mice lung tissues or A549 cells were obtained and added to RIPA lysis solution to break up the tissues/cells, then centrifuged at 12,000 r/min for 5 min at 4 °C. Then collected supernatant and measured the protein concentration using the BCA method, adjusted to the same concentration with PBS and then added Loading Buffer and heating at 100 °C for 5 min. 20 μg of protein sample was separated by 10% sodium dodecyl sulfate-polyacrylamide gel electrophoresis (SDS-PAGE) and transferred to a polyvinylidene fluoride (PVDF) membrane. The PVDF membrane was cut into different strips according to molecular weight, then blocked with 5% skimmed milk powder for one hour. After that, β-actin, TGF-β1, COL2A1, AKT, p-AKT, mTOR, and p-mTOR primary antibodies were added to the corresponding bands, respectively, diluted with 5% skimmed milk powder, all at a concentration of 1:1000, incubated overnight at 4 °C, and then washed three times with TBST, 10 min each time. Incubated for 2 h on a shaker using a 5% skimmed milk powder solution containing horseradish peroxidase-labeled secondary antibody (1:1000 dilution), washed three times for 10 min each using TBST; visualized using the ECL Advanced Western Blot Detection Kit and images captured using a gel imaging system (BIO-RAD, Hercules, CA, USA); quantitative analysis using Image J. 

### 3.11. Statistical Analyses

All the statistics are carried out using the GraphPad Prism Version 6.02 version of GraphPad Prism (GraphPad Software, San Diego, CA, USA). In this study, the data were processed by one-way ANOVA and post-hoc Turkey. Statistical significance was determined when * *p* < 0.05, ** *p* < 0.01 and *** *p* < 0.001.

## 4. Conclusions

In this study, we successfully extracted fucoidan (CCP) from Costria costata, a sulfated polymer of galactose and fucose. The molecular weight of CCP is about 150 kDa, and the sulfate group content is 18.54%. Our study firstly evaluated the anti-idiopathic fibrosis activity of CCP, and fund CCP exhibited anti-EMT activity in TGF-β1-induced A549 cells. Its anti-EMT effect was regulated through both the TGF-β/Smad signaling pathway and the PI3K/AKT/mTOR signaling pathway. In addition, CCP exhibited potent anti-pulmonary fibrosis activity in vivo by reducing collagen deposits and down-regulated the expression of TGF-β1 and IL-6. Therefore, this study proposed a new trehalose gum for treating pulmonary interstitial fibrosis. However, the anti-pulmonary fibrosis mechanism of CCP still needs further investigation.

## Figures and Tables

**Table 1:** CCP chemical composition analysis. (%, dry weight) and monosaccharide composition (molar ratio, Fuc = 1).

Yield (%)	Sulfate Content (%)	Fucose Content (%)	GlcA Content (%)	Protein Content (%)	Molecular Weight (Da)	Monosaccharide Composition						
						Man	Rha	GlcA	Glc	Gal	Xyl	Fuc
6.90	18.54	14.85 ± 0.13	13.20 ± 0.26	1.38 ± 0.06	149,846	0.244	0.111	0.073	0.042	0.906	0.03	1

## Data Availability

Not applicable.

## Nomenclature

CCP	*Costaria costata* polysaccharide
TGF-β1	Transforming growth factor-β1
EMT	Epithelial-mesenchymal transition
COL2A1	Collagen, type II, alpha 1
BLM	Bleomycin
IPF	Idiopathic pulmonary fibrosis
ECM	Extracellular matrix
PMP	1-phenyl-3-methyl-5-pyrazolone
DMSO	Dimethyl sulfoxide
MTT	3-(4, 5-dimethylthiazol-2-yl)-2, 5-diphenyltetrazolium bromide
ELISA	Enzyme-linked immunosorbent assay
IL-6	Interleukin-6
TPA	Total Phenol Assay
